# Molecular Insight into Acute Limb Ischemia

**DOI:** 10.3390/biom14070838

**Published:** 2024-07-11

**Authors:** Davide Costa, Nicola Ielapi, Paolo Perri, Roberto Minici, Teresa Faga, Ashour Michael, Umberto Marcello Bracale, Michele Andreucci, Raffaele Serra

**Affiliations:** 1 Department of Medical and Surgical Sciences, Magna Graecia University of Catanzaro, 88100 Catanzaro, Italy; davide.costa@unicz.it (D.C.); nicola.ielapi@uniroma1.it (N.I.); 2 Interuniversity Center of Phlebolymphology (CIFL), “Magna Graecia” University, 88100 Catanzaro, Italy; 3 Department of Public Health and Infectious Disease, “Sapienza” University of Rome, 00185 Rome, Italy; 4 Department of Vascular and Endovascular Surgery, Annunziata Hospital, 1 Via Migliori, 87100 Cosenza, Italy; p.perri@aocs.it; 5 Department of Experimental and Clinical Medicine, Magna Graecia University of Catanzaro, 88100 Catanzaro, Italy; miniciroberto@gmail.com; 6 Department of Health Sciences, Magna Graecia University of Catanzaro, 88100 Catanzaro, Italy; teresa_faga@yahoo.it (T.F.); ashourmichael@yahoo.com (A.M.); 7 Department of Public Health, University Federico II of Naples, 80131 Naples, Italy; umbertomarcello.bracale@unina.it

**Keywords:** acute limb ischemia, ALI, arterial thrombosis, arterial embolism, ischemia/reperfusion, biomarkers, inflammation, biomolecules, cytokines

## Abstract

Acute limb ischemia (ALI) is defined as a sudden reduction in blood flow to a limb, resulting in cessation of blood flow and, therefore, cessation of the delivery of nutrients and oxygen to the tissues of the lower limb. Despite optimal treatment to restore blood flow to ischemic tissues, some patients may suffer from ischemia/reperfusion (I/R) syndrome, the most severe complication after a revascularization procedure used to restore blood flow. There are multiple molecular and cellular factors that are involved in each phase of ALI. This review focuses firstly on molecular and cellular factors of arterial thrombosis, highlighting the role of atherosclerotic plaques, smooth muscle cells (SMCs), and cytokine which may alter key components of the extracellular matrix (ECM). Then, molecular and cellular factors of arterial embolism will be discussed, highlighting the importance of thrombi composition. Molecular and cellular factors of ischemia/reperfusion syndrome are analyzed in depth, highlighting several important mechanisms related to tissue damage, such as inflammation, apoptosis, autophagy, necrosis, and necroptosis. Furthermore, local and general complications of ALI are discussed in the context of molecular alterations. Ultimately, the role of novel biomarkers and targeted therapies is discussed.

## 1. Introduction

Acute limb ischemia (ALI) is defined as a sudden reduction in limb perfusion, resulting in the cessation of blood flow and delivery of nutrients and oxygen to tissues of lower limbs. ALI occurs most often in patients with pre-existing peripheral arterial disease (PAD), and, if the obstruction is not resolved, patients with PAD will experience new or worsening signs and symptoms of ischemia, putting limb viability at risk. On the other hand, when ALI occurs in individuals with normal underlying arterial circulation, unlike chronic limb ischemia, ischemic damage can develop rather rapidly, over minutes to hours, and this can lead to gangrene of the limbs, resulting in the need for amputation. The differences between healthy arteries and arteries of PAD patients are caused by the presence of collateral blood flow that develops to bypass an occlusion in PAD patients. In particular, enlarged collateral vessels, which form in chronically poorly perfused tissue of PAD patients, help to maintain a minimum blood supply to tissues of lower limbs despite occlusion in these patients. But, in healthy vasculature there is no opportunity for collateral flow to develop, and in the case of ALI, the clinical consequences are obviously worse. The most common causes of ALI include arterial thrombosis or its progression or embolisms in PAD arteries, embolisms in the heart, arterial dissection, thrombosis and/or peripheral aneurisms of lower limbs, trauma, or occlusion of a previous bypass graft [1,2,3].

ALI occurs at a rate of approximately 14 cases per 100,000 people per year, and despite this low frequency, it has important morbidity and mortality. In fact, acute limb ischemia determines a 30-day amputation rate of 10–30% and a mortality rate between 9 and 25% [2,4,5].

Currently, the gold standard treatment for acute limb ischemia is urgent or emergent revascularization. Despite optimal treatment to restore blood flow to the ischemic tissues, several patients may suffer from ischemia/reperfusion (I/R) syndrome, which is the most serious adverse complication of revascularization [6].

I/R syndrome is essentially composed of two components: a local component, determined by the activation of cellular and molecular factors related to inflammation with subsequent formation of reactive oxygen species (ROS); ion dysregulation cell death and increased tissue compression with capillary occlusion (compartment syndrome); and an increase in ischemic lesions; and a systemic component, also called post-reperfusion syndrome (PRS), with secondary failure of organs and tissues, such as liver and kidney; acute respiratory distress syndrome (ARDS); and multi-organ failure [6,7].

There are multiple molecular and cellular factors that cause an atherosclerotic plaque that has developed over many years to suddenly become unstable and cause an acute thrombotic occlusion with subsequent ALI onset. There are also other molecular and cellular factors that sustain I/R syndrome [3,6,7]. The aim of this article was to review the most up-to-date articles dealing with molecular determinants of ALI and its complications, to help researchers and physicians to better understand these phenomena and to better tailor actual treatments that facilitate precision medicine.

In conducting this review, the searched libraries were Web of Science, Scopus, ScienceDirect, and Medline. We set no time limits.

The keywords used, with various combinations, were “acute limb ischemia”, “atherothrombosis”, “ischemia/reperfusion syndrome”, “post-reperfusion syndrome”, “compartment syndrome”, “molecular”, “cellular”, “pathophysiology”, and “histopathology”.

Table 1 summarizes relevant articles found after literature search.

## 2. Molecular and Cellular Factors of Arterial Thrombosis

**Figure 1:**
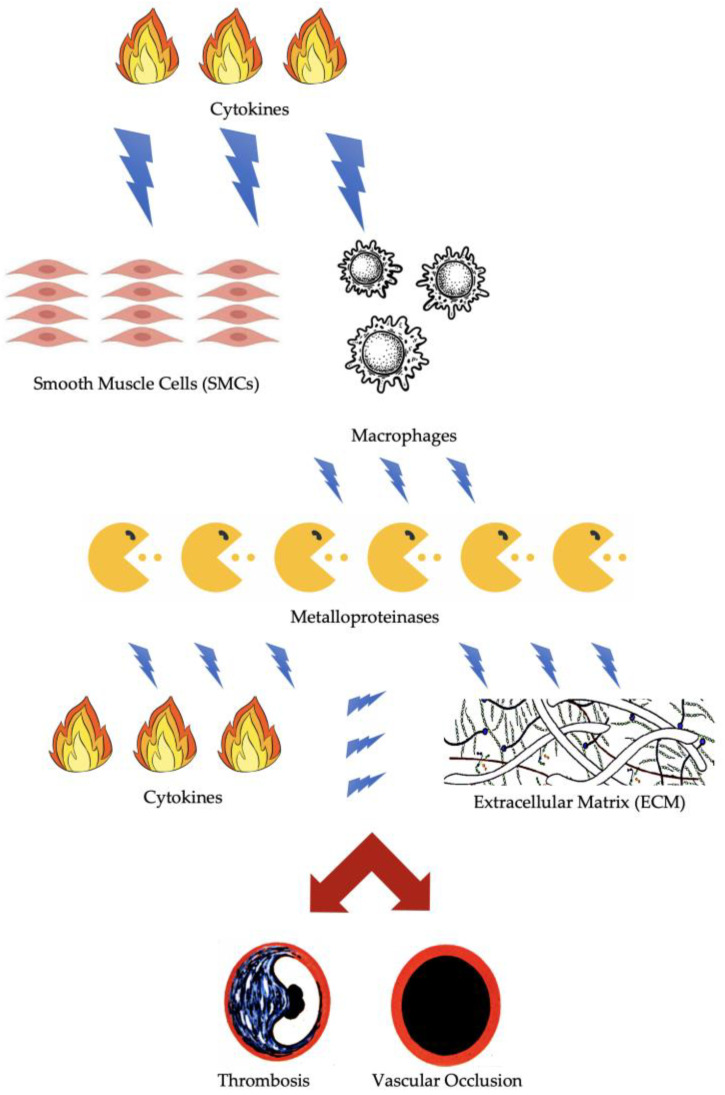
Pathophysiology of thrombosis and vascular occlusion.

For several reasons, atherosclerotic plaques that have developed over time can suddenly become unstable and cause acute thrombotic occlusion. Histological examination may be useful to understand these phenomena. During the growth of atherosclerotic plaques, smooth muscle cells (SMCs) are stimulated by a cytokine gradient to migrate from the media to the intima, where they produce key components of the extracellular matrix (ECM) and form a fibrous cap that stabilizes the plaque and the necrotic core [7,8,9]. Macrophages located under this cap digest the accumulated oxidized low-density lipoprotein (LDL) and release cytokines and metalloproteinases (MPs) that enhance the inflammatory response. The presence of a thin fibrous cap and a highly necrotic and decayed core containing sprouting capillaries within the hypoxic plaque induces microbleeds and further recruitment of inflammatory cells. The gradual degradation of SMCs causes plaque destabilization, eventually leading to rupture of the fibrous cap with subsequent thrombosis and vascular occlusion [10,11] (Figure 1). Whether a plaque ruptures during physical exercise or other mechanical stress depends on the exact composition of its structure. Given that many inflammatory cells are mainly located in the shoulder regions of the plaque, it is likely that the fibrous cap will rupture in these locations, even at rest. This is due to accelerated degradation of structural proteins, which leads to instability of the plaque edges. In contrast, rupture of the center of an atheroma is mainly caused by calcification, which reduces the elasticity of the fibrous cap [8,12,13].

## 3. Molecular and Cellular Factors of Arterial Embolism

Arterial embolism occurs when a tissue mass travels through the circulation and eventually lodges in a distal artery, obstructing blood flow. This blockage can lead to local ischemia. Arterial embolism may arise from the heart or other proximal diseased large arteries, such as aneurysms with intraluminal thrombus formation. The embolus may travel to extremities, thus causing limb ischemia. This is primarily observed in patients suffering from heart or artery diseases such as arrhythmia, aortic aneurysm, or dissection [8]. Furthermore, most emboli occur in patients with significant underlying disease, those of advanced age, or those in the surgical and critical-care population. Arterial embolism has many similarities to arterial thrombosis in PAD patients, but there are also important differences. Perhaps the most important difference between the two processes is the severity of symptoms. Because arterial thrombosis in PAD patients is an insidious process that manifests as ischemia in its later stages, patients often have time to develop collateral blood flow to the ischemic area. However, people with embolic ischemia usually do not establish collateral circulation and, therefore, often experience more sudden and severe symptoms that lead to imminent limb loss [14]. Histological analyses show that principal components of arterial thrombi include fibrin, platelets, red blood cells (RBCs), leukocytes, and neutrophil extracellular traps (NETs). It is possible that thrombi with a particular composition are more prone to embolize. In fact, RBCs and fibrin content seem to be higher in embolizing thrombi [15].

## 4. Molecular and Cellular Factors of Acute Limb Ischemia

ALI is responsible for local tissue alteration of the affected limb, and for molecular and cellular dysfunction, such as mitochondrial alterations that will also lead to more severe manifestations related to I/R injury [7]. Specifically, mitochondrial functions include adenosine triphosphate (ATP) production, ROS production, degradation, metabolite synthesis, catabolism, and regulation of apoptosis. Hypoxia in skeletal muscle depletes ATP, a major energy source resulting from oxidative phosphorylation. Mammalian skeletal muscle has different fiber types that can withstand different levels of hypoxia. In the lower extremities, type I muscle fibers (red slow-twitch muscle fibers) in the front of the leg are more susceptible to hypoxia than type II muscle fibers (white fast-twitch fibers) in the gastrocnemius muscle [6,15,16]. At the cellular level, mitochondrial dysfunction plays an important role in the development of I/R syndrome. In fact, I/R events have been shown to alter mitochondrial capacity, causing mitochondrial fragmentation and leading to apoptosis [17]. A study highlighted that the muscle showed apoptosis in these cells after 5 h of hypoxia [18]. Under hypoxic conditions, xanthine dehydrogenase (D type) converts to xanthine oxidase (O type), thereby increasing ROS production. These excess ROS levels can cause cellular oxidative stress, leading to protein carboxylation, lipid oxidation, and deoxyribonucleic acid (DNA) damage. In addition, ATP deficiency induces the expression of B-cell lymphoma 2 (Bcl-2), Bcl-2-like protein 4 (Bax), and Bcl-2-related cell death agonist protein (Bad) in the mitochondrial membrane, leading to mitochondrial swelling, and mitochondrial fission. Furthermore, an increase in intracellular Ca^2+^ levels under hypoxic conditions allows the mitochondrial permeability transition pore (mPTP) to switch from a transiently open to a permanently open state. Opening of the mPTP allows ROS and cytochrome c to escape into the cell matrix, causing Ca^2+^ influx, mitochondrial swelling and membrane rupture, and induction of intracellular apoptosis [6,19,20,21,22]. Furthermore, ischemia induces the expression of several proinflammatory gene products such as leukocyte adhesion molecules, cytokines and bioactive substances such as endothelin, and thromboxane A2, thereby suppressing the release of nitric oxide synthase (NOS), thrombomodulin, and bioactive substances (prostacyclin). Moreover, hypoxia promotes both transcriptional and non-transcriptional pathways, and distinct pathways exist between tissue responses to hypoxia and acute inflammation [23].

## 5. Molecular and Cellular Factors of Ischemia/Reperfusion Syndrome

I/R injury is a process of sustained or accelerated local damage that occurs in previously ischemic body tissues after blood supply is restored. The pathophysiology of I/R injury begins with a hypoxic event in the endothelium, which not only impairs vascular barrier function but also affects the activity of polymorphonuclear leukocytes (PMNs). I/R injury also triggers complement activation and the production of important inflammatory mediators that can upset the balance of vascular homeostasis. The anaphylatoxin C5a can increase the inflammatory response through the production of cytokines such as monocyte chemotactic protein 1 (MCP-1), tumor necrosis factor α (TNF α), interleukin-1 (IL-1), and interleukin 6 (IL-6), which are also involved in the acute-phase protein response [23].

Skeletal muscles of the limbs are highly sensitive to ischemia, and since muscle constitutes most of the limb tissue, muscle damage is clearly the most important aspect of limb reperfusion syndrome. Moreover, the degree of skeletal muscle damage directly correlates with the severity and duration of ischemia. Changes in the microcirculation lead to increased vascular permeability to plasma proteins and determine interstitial edema depending on the muscle mass involved in the ischemic injury. The inflammatory response may remain mainly local or may be both local and systemic [23,24].

Much research has shown that I/R injury is associated with endothelial cells (ECs) and ECM activation. ECs trigger both the complement and coagulation systems and stimulate the recruitment of proinflammatory cells, which gradually leads to the no-reflow phenomenon where a localized ischemic area does not receive blood flow again after blood flow has been restored. Dysfunction of both sodium-potassium ATPase and calcium-sodium exchanger is also implicated. Increased concentrations of free calcium interact with actin, myosin, and cellular proteases, leading to necrosis of skeletal muscle fibers. These phenomena are enhanced by reperfusion. Local complications of I/R injury may also result in severe functional failure of amputated or salvaged limbs. Massive local I/R injury often leads to systemic complications, a phenomenon called PRS. Release of myoglobin, potassium, lactate, and microthrombi from injured skeletal muscle into the systemic circulation can cause renal failure, arrhythmias, and, ultimately, death [7,24,25,26,27,28,29].

As several studies have demonstrated, in the lower limbs, an ischemic duration of approximately 5 h represents a critical time for cell death and the progression of the no-reflow phenomenon [23,24].

Several functional and mechanical changes explain the appearance of the no-reflow phenomenon, such as the formation of interstitial edema caused by the increase in capillary permeability that also causes the compressive phenomena of passive vasoconstriction, and microvascular spasm (active vasoconstriction). All this leads to transcapillary fluid filtration, which results in interstitial edema that physically compresses the capillaries, promoting the no-reflow phenomenon. Another mechanism for no-reflow could be an altered production of vasoactive mediators, which could manifest as an imbalance between vasodilators and vasoconstrictors, resulting in local blood-flow insufficiency [24,30,31].

From a cellular point of view, cell swelling and rupture, and cell death by apoptotic, autophagic, necrotic, or necroptotic mechanisms are pivotal in I/R injury [32].

The apoptotic machinery has two pathways, the “extrinsic” and “intrinsic”, but there are also important interactions between these two pathways. The “extrinsic” pathway activates receptors such as Fas, TNF α, and targeting TNF-related apoptosis-inducing ligand (TRAIL), which leads to trimerization and recruitment of several proteins containing death domains, such as FAS-associated death domain (FADD) protein and tumor necrosis factor receptor type 1-associated DEATH domain (TRADD) protein, to the receptor complex. This death-inducing signaling complex activates the protease caspase-8, which in turn cleaves and activates caspase-3. Caspase-3 acts by proteolyzing many cellular proteins. More specifically, in the “intrinsic” signaling pathway, I/R induces the translocation and integration of death-promoting members of the Bcl-2 protein family (e.g., Bax and Bak) into the outer mitochondrial membrane. These proteins can permeabilize the outer membrane, allowing the release of pro-apoptotic proteins, notably cytochrome c, Smac/Diablo, Omi/HtrA2, and endonuclease G (endoG), from the intermembrane space. Cytochrome c binds to the cytosolic protein apaf1, and the resulting “apoptosome” activates caspase-9 and the β3 protease system. Smac/Diablo and Omi/HtrA2 activate caspases by sequestering and digesting caspase-inhibitory proteins, respectively, while EndoG mediates DNA fragmentation [32,33,34,35].

Autophagy is the main mechanism of cells to dispose of old or damaged organelles and protein aggregates, performing a ‘housekeeping function’. Morphologically, autophagy begins with the expansion of an insulating membrane or phagophore around the cellular compartment/organelle to be processed. The membrane then completely encapsulates the component to form a vesicle, the autophagosome, which then fuses with a lysosome to degrade the encapsulated material. Like apoptosis, autophagy is tightly regulated and mediated by specific pathways that include the actions of rapamycin (mTOR), a complex consisting of vps34, vps15, and a class III PI3K called Beclin-1. This complex then recruits Atg12, Atg5, and Atg8, and is also known as microtubule-associated protein 1A/1B light chain 3 (LC3), which is essential for membrane elongation and autophagosome completion. The now-completed fusion of autophagosomes with lysosomes is mediated by the small GTPase Rab7 and the lysosome-associated membrane protein 2 (LAMP2) [32,36,37,38].

Necrosis is characterized histologically by swelling of the cell and its organelles, mitochondrial dysfunction, lack of nuclear fragmentation, rupture of the plasma membrane, and leakage of intracellular contents. Compared with the programmed mechanism of apoptosis and autophagy, necrosis is thought to be a random, uncontrolled process resulting in the random death of cells in response to intense stress, but in I/R injury, we can talk about the of the concept of programmed necrosis, also termed necroptosis. In fact, it is known that cell stress or death-receptor activation activates, in turn, some serine/threonine kinases called receptor interacting proteins (RIPs). RIP1 and RIP3 are considered the main mediators of necrosis, linked also to increased ROS production, and TNF α-related mechanisms [32,39].

## 6. The Role of Biomarkers in Clinical Practice

In the context of precision medicine, biochemical markers of ischemia/reperfusion injury have been of long-standing interest in the vascular-surgery research field. ALI has a typical clinical scenario in which these markers seem to be particularly relevant. The use of biomarkers to predict, pre- or perioperatively, which patients will not be successful after limb salvage attempts or will have a worse functional outcome after salvage, is of pivotal interest [40,41]. As inflammation is one of the key concepts in the pathophysiology of ALI, biomarkers that are related to inflammation may be good candidates for predicting outcomes [23]. The neutrophil to lymphocyte ratio (NLR) is an inexpensive test that reflects systemic inflammation, and it was found to be related to several vascular diseases [40,41,42]. For example, several studies have shown that an NLR > 5 in ALI patients is associated with higher mortality and morbidity rates, suggesting to surgeons that they be timelier and more aggressive in attempts to revascularize lower limbs and, perhaps, that they use adjunct treatments such as prophylactic fasciotomies, or catheter-based or shunt-delivered pharmacotherapies, such as oxygen-radical scavengers or mitochondrial stabilizers [40,43,44,45].

As I/R injury is related to ECs and ECM activation, which is regulated mainly by matrix metalloproteinases (MMPs), that may also modulate the actions of inflammatory cytokines. A study [7] showed significant association between critical biochemical parameter levels, which indicate damaged organs of ALI patients, and high blood levels of MMP-1, -2, -3, and -9. Another molecule found in this study was neutrophil gelatinase-associated lipocalin (NGAL), which generally enhances the activity of MMP-9 by the formation of the NGAL/MMP-9 complex that protects MMP-9 from proteolytic degradation maintaining its activity for a longer time [46,47].

MMPs are generally related to a wide range of vascular disease and may serve as biomarkers of onset, treatment selection, response to treatment, follow up, and relapses [48].

In PAD patients with severe limb ischemia (SLI), a study showed that some inflammatory biomarkers may predict major outcomes such as ALI and amputation. IL-6 and Interferon Gamma-Induced Protein 10 (IP-10) seem able to predict the occurrence of adverse and major events in this patient population [49]. IL-6 is a cytokine that stimulates the production of acute-phase reactants (APRs), including CRP and fibrinogen, that in turn can induce a hypercoagulable state in the body, thus leading to thrombosis and ALI [50]. IP-10 is known to act with several thrombo-inflammatory mechanisms [51].

Moreover, muscle ischemia and subsequent I/R injury result in massive release of Mb and creatine kinase (CK) by muscle cells, both of which are considered surrogates for muscle damage caused by ischemia. Thus, elevated serum Mb and CK levels are associated with the development of rhabdomyolysis, reflecting the extent of muscle damage. Mb is a metabolite directly responsible for acute kidney injury (AKI) after revascularization in ALI. Indeed, Mb-induced kidney damage results from the deposition of Mb in the distal tubules, leading to direct nephrotoxicity through tubular obstruction as well as liposomal peroxidation and inflammation in the context of aciduria [40]. Although Mb is perhaps the more intuitive marker of skeletal ischemia in the setting of ALI, CK seems to be more sensitive to muscle damage; elevated plasma CK levels at a patient’s admission seems to be predictive of major amputation in the setting of ALI [40].

After endovascular treatment for ALI, elevated high-sensitivity troponin T (hsTnT) seems to be associated with worse in-hospital outcomes such as major amputation and mortality. High-sensitivity troponin T (hsTnT) is one of the most accepted specific biomarkers of cardiac damage, which are released in conditions of cardiomyocyte stress and/or injury. The mechanisms linking PAD and the cardiac release of hsTnT are likely multifactorial, probably related to the high coexistence of PAD and CAD, but this has not yet been fully elucidated [52].

## 7. Discussion

ALI is a vascular emergency characterized by important symptoms, signs, and loss of function, followed by gangrene of the affected limb. The extent of tissue damage depends on the reach of the collateral network. Severe tissue destruction not only affects the affected limb, but also has consequences that endanger the entire body. From a pathophysiological point of view, ALI involves a complex network of control mechanisms that become dysregulated, with many of them being particularly difficult to counteract [8].

In ALI, there is a shift of fluid into the tissues, especially during the reperfusion phase, with a concomitant narrowing of the vascular bed, which becomes unresponsive to vasorelaxant factors. This process generally leads to an exacerbation of the cycle in which increased peripheral resistance causes further oxygen deprivation. This entire process is accompanied by a massive outgrowth of inflammatory cells, which release cytokines and increase the inflammatory response. Acute hypoxia can be life-threatening, as all cells depend on the energy generated by aerobic respiration. Aerobic processes involved in the generation of adenosine triphosphate (ATP), such as the electron transport chain and oxidative phosphorylation in mitochondria, are affected first, disrupting the integrity of cellular respiration. This results in irreparable damage to cell membranes due to electrolyte imbalance [8,15,16,17].

When I/R injury occurs, oxygen itself causes further damage to the cell and initiates apoptosis in surrounding cells. ROS are formed, causing severe damage to cell membranes and DNA. However, the increase in tissue stress caused by this process may be temporary, as free radical scavengers can mitigate the damage. Finally, considering the above, acute ischemia followed by reperfusion is a process that causes acute tissue damage combined with end-organ loss of function [8].

The reperfusion phase also allows the recruitment of activated neutrophils, resulting in the release of numerous inflammatory signals that lead to the destruction of the endothelium, further compromising tissue perfusion [40].

**Figure 2:**
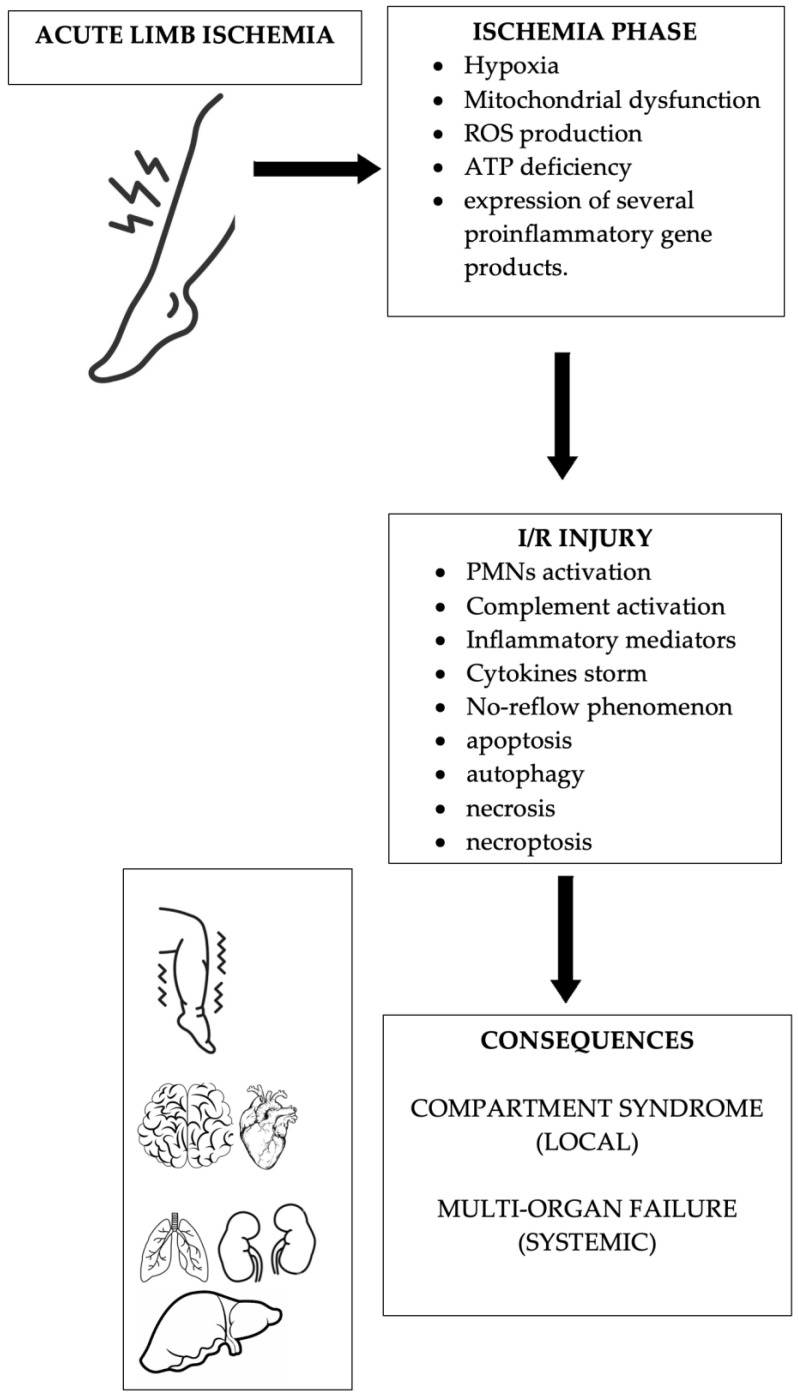
Main pathophysiological mechanisms of ALI and I/R injury. ROS = reactive oxygen species; ATP = adenosine triphosphate; PMN = polymorphonuclear leukocytes.

The main pathophysiological mechanisms of ALI and I/R injury are shown in Figure 2.

In recent decades, various interventions have been proposed to reduce limb muscle I/R injury. These include pharmacological and non-pharmacological treatments that have been described in both basic and clinical research. Antioxidants and anti-inflammatory drugs have been used to prevent I/R injury. Non-pharmacological interventions such as ischemic preconditioning of controlled reperfusion have also been used, especially in clinical trials. Unfortunately, there is currently no consensus on therapeutic interventions for patients with I/R injury of lower limbs [6].

Further research is focused on identifying new biomarkers, new pharmacological therapies, and molecular regulators that can influence and predict outcome. Measures used to prevent injury are presented by allopurinol and superoxide dismutase, which are designed to bind specific molecules of I/R injury, and by mitochondrial transition permeability pore regulators such as cyclosporine A, bongkrekic acid, and alisporivir that target mitochondrial dysfunction that is responsible for irreversible cellular and tissue damage [40,53,54]. Other promising strategies include targeting neutrophil activity and adhesion neutralization through antibody targeting against intercellular adhesion molecule-1 (ICAM-1) and P-selectin [40,55].

Recent evidence has shown that several gaseous molecules, such as hydrogen and carbon monoxide, have significant anti-inflammatory, anti-apoptotic, and antioxidant protective effects on cells and tissues in ALI patients. The use of medical gases for oxidative stress therapy seems to be particularly promising. Medical gases can be administered to patients by inhalation using several devices such as nasal cannula, a ventilator circuit, or a face mask [56,57,58].

Biomarkers may be used in the future, even for treatment purposes and clinical decision-making; for example, pharmacotherapy based on the manipulation of specific cytokines, found elevated in biomarkers assessment, has been successfully tested in animal models of ischemia/reperfusion injury. Many approaches have been investigated, suggesting that preconditioning of tissues with cytokines or compounds designed to inhibit the activation of proinflammatory transcription factors such as nuclear factor kappa-light-chain-enhancer of activated B cells (NF-κB), for example, during surgery, may be very useful. This may reduce tissue damage caused by reactive oxygen species and prevent the development of proinflammatory cytokines during subsequent reperfusion. Another approach is to target specific cytokines after reperfusion. Moreover, treatment of perfused tissues with agents targeting chemokines and adhesion molecules may limit leukocyte adhesion/recruitment and provide benefits against I/R injury. Indeed, combination therapy, which arises from targeted decision-making, offers the greatest chance of success in treating I/R injury in many clinical situations [7,59].

The limitations of this review include its narrative nature, as narrative reviews may not always provide an evidence-based synthesis for focused questions, nor do they offer definitive answers. The findings and the related interpretations may also vary depending on the authors’ team or the context of the analysis. Moreover, narrative reviews lack a pre-defined methodology, making reproduction of the authors’ course impossible and preventing systematic checks for biases. On the other hand, narrative reviews can be performed quickly and may include non-peer-reviewed sources of information. Narrative reviews have been criticized for rarely employing peer-reviewed methodologies, or duplicate curation of evidence, and for often failing to disclose study inclusion criteria. Despite these limitations, narrative reviews remain frequent within the literature, as they offer a breadth of literature coverage and the flexibility to deal with evolving knowledge and concepts [60].

Furthermore, in the near future, effective prediction models using targeted biomarkers could be instrumental in improving risk-stratification in ALI patients, and thus may help clinical decision-making for tailored treatment.

## Figures and Tables

**Table 1:** Literature search at a glance.

Author	Article Title	Topics
Simon, F., et al. [3]	Acute Limb Ischemia-Much More Than Just a Lack of Oxygen.	ALI: mechanisms of acute tissue damage combined with end-organ loss of function.
Apichartpiyakul, P., et al. [6]	Mechanisms and Interventions on Acute Lower Limb Ischemia/Reperfusion Injury: A Review and Insights from Cell to Clinical Investigations.	Cellular mechanisms of ALI and I/R injury.
de Franciscis, S., et al. [7]	Biomarkers in post-reperfusion syndrome after acute lower limb ischaemia.	Biomarkers in I/R injury.
Lorentzen, L. G., et al. [8]	Proteomic analysis of the extracellular matrix of human atherosclerotic plaques shows marked changes between plaque types.	Plaque Destabilization: plaque compositions and plaques types in atherosclerosis.
Koga, J., et al. [9]	Crosstalk between macrophages and smooth muscle cells in atherosclerotic vascular diseases.	Plaque Destabilization: role of macrophages and smooth muscle cells (SMCs) in atherosclerotic lesions and plaque destabilization.
Virmani, R., et al. [10]	Lessons from sudden coronary death: a comprehensive morphological classification scheme for atherosclerotic lesions.	Plaque Destabilization: morphological insight of atherosclerotic plaques.
Moreno, P. R., et al. [11]	Plaque neovascularization: defense mechanisms, betrayal, or a war in progress.	Plaque Destabilization: role of plaque neovascularization.
Burke, A. P., et al. [12]	Plaque rupture and sudden death related to exertion in men with coronary artery disease.	Plaque Destabilization: mechanims of plaque rupture.
Tedgui, A., et al. [13]	Apoptosis as a determinant of atherothrombosis.	Apoptosis: role in atherothrombosis.
Lyaker, et al. [14]	Arterial embolism.	Arterial embolism: pathophysiology.
Alkarithi, G., et al. [15]	Thrombus Structural Composition in Cardiovascular Disease.	Arterial embolism: relationship with thrombus structure.
Lejay, A., et al. [16]	Mitochondria: mitochondrial participation in ischemia-reperfusion injury in skeletal muscle.	Cellular factors and components related to ischemia: the role of mitochondria.
Lindsay, T. F., et al. [17]	The effect of ischemia/reperfusion on adenine nucleotide metabolism and xanthine oxidase production in skeletal muscle.	Cellular factors and components related to ischemia: the role of cellular metabolism.
Youle, R. J., et al. [18]	Mitochondrial fission, fusion, and stress.	Cellular factors and components related to ischemia: the role of mitochondria.
Wilson, H. M., et al. [19]	Can Cytoprotective Cobalt Protoporphyrin Protect Skeletal Muscle and Muscle-derived Stem Cells From Ischemic Injury?	Cellular factors and components related to ischemia: the role of muscle tissues.
Chambers, D. E., et al. [20]	Xanthine oxidase as a source of free radical damage in myocardial ischemia.	Cellular factors and components related to ischemia: mechanisms of damage.
Gao, X., et al. [21]	Glycine-nitronyl nitroxide conjugate protects human umbilical vein endothelial cells against hypoxia/reoxygenation injury via multiple mechanisms and ameliorates hind limb ischemia/reperfusion injury in rats.	Cellular factors and components related to ischemia: mechanisms of damage.
McCully JD, et al. [22]	Differential contribution of necrosis and apoptosis in myocardial ischemia-reperfusion injury.	Apoptosis: role in I/R injury.
Serra, R., et al. [23]	Fatal early peripheral post-reperfusion syndrome and the role of cutaneous signs.	Cutaneous signs in post-reperfusion syndrome.
Nanobashvili, J., et al. [24]	Development of ‘no-reflow’ phenomenon in ischemia/reperfusion injury: failure of active vasomotility and not simply passive vasoconstriction.	I/R injury: the role of No-reflow’ phenomenon.
Chappell, D., et al. [25]	Sevoflurane reduces leukocyte and platelet adhesion after ischemia-reperfusion by protecting the endothelial glycocalyx.	I/R injury: the role of endothelial glycocalyx.
Abela, C. B., et al. [26]	Clinical implications of ischaemia-reperfusion injury.	I/R injury: pathophysiology.
Szijártó, A., et al. [27]	Rapidly progressing fatal reperfusion syndrome caused by acute critical ischemia of the lower limb.	I/R injury: and severity of symptoms.
Cruz, C. P., et al. [28]	Major lower extremity amputations at a Veterans Affairs hospital.	I/R injury and severity of symptoms.
Klausner, J. M., et al. [29]	Oxygen free radicals mediate ischemia-induced lung injury.	I/R injury and severity of symptoms.
Allen, D. M., et al. [30]	Pathophysiology and related studies of the no reflow phenomenon in skeletal muscle.	I/R injury: the role of No-reflow’ phenomenon.
Gute, D. C., et al. [31]	Inflammatory responses to ischemia and reperfusion in skeletal muscle.	I/R injury: the role of inflammation.
Kalogeris, T., et al. [32]	Cell biology of ischemia/reperfusion injury.	I/R injury: cellular mechanisms.
Broughton, B. R., et al. [33]	Apoptotic mechanisms after cerebral ischemia.	Apoptosis: cellular mechanisms.
Kroemer, G., et al. [34]	Mitochondrial membrane permeabilization in cell death.	Cellular factors and components related to ischemia: the role of mitochondria.
Whelan, R. S., et al. [35]	Cell death in the pathogenesis of heart disease: mechanisms and significance.	Apoptosis: cellular mechanisms.
Gottlieb, R. A., et al. [36]	Autophagy during cardiac stress: joys and frustrations of autophagy.	Autophagy and I/R injury.
He, C., et al. [37]	Regulation mechanisms and signaling pathways of autophagy.	Autophagy and I/R injury.
Levine, B., et al. [38]	Autophagy in the pathogenesis of disease.	Autophagy and I/R injury.
Morgan, M. J., et al. [39]	TNFalpha and reactive oxygen species in necrotic cell death.	Autophagy and cytokines
Watson, J. D., et al. [40]	Biochemical markers of acute limb ischemia, rhabdomyolysis, and impact on limb salvage.	Biomarkers in I/R injury.
Ielapi, N., et al. [41]	Precision Medicine and Precision Nursing: The Era of Biomarkers and Precision Health.	Biomarkers and Precision Medicine.
Serra, R., et al. [42]	Neutrophil-to-lymphocyte Ratio and Platelet-to-lymphocyte Ratio as Biomarkers for Cardiovascular Surgery Procedures: A Literature Review.	Biomarkers: role of inflammation biomarkers.
Bhutta, H., et al. [43]	Neutrophil-lymphocyte ratio predicts medium-term survival following elective major vascular surgery: a cross-sectional study.	Biomarkers: role of inflammation biomarkers.
Chan, C., et al. [44]	Neutrophil-lymphocyte ratio as a prognostic marker of outcome in infrapopliteal percutaneous interventions for critical limb ischemia.	Biomarkers: role of inflammation biomarkers.
Taurino, M., et al. [45]	Neutrophil-to-Lymphocyte Ratio Could Predict Outcome in Patients Presenting with Acute Limb Ischemia.	Biomarkers: role of inflammation biomarkers.
Busceti, M.T., et al. [46]	Pulmonary embolism, metalloproteinsases and neutrophil gelatinase associated lipocalin.	Biomarkers: role of inflammation biomarkers.
Costa, D., et al. [47]	Vascular Biology of Arterial Aneurysms.	Biomarkers: role of inflammation biomarkers.
Costa, D., et al. [48]	Metalloproteinases as Biomarkers and Sociomarkers in Human Health and Disease.	Biomarkers: role of inflammation biomarkers.
Gremmels, H., et al. [49]	A Pro-Inflammatory Biomarker-Profile Predicts Amputation-Free Survival in Patients with Severe Limb Ischemia.	Biomarkers: role of inflammation biomarkers.
Hasan, S. A., et al. [50]	Acute Limb Ischemia: A Rare Complication of COVID-19.	Biomarkers: role of inflammation biomarkers.
Grosse, G. M., at al. [51]	Circulating Cytokines and Growth Factors in Acute Cerebral Large Vessel Occlusion-Association with Success of Endovascular Treatment.	Biomarkers: role of inflammation biomarkers.
